# Microcatheter-Assisted Stenting of a Tortuous Obstructed Vertical Vein in a Case of Obstructive Total Anomalous Pulmonary Venous Connection

**DOI:** 10.7759/cureus.86046

**Published:** 2025-06-15

**Authors:** Aamir Rashid, Qayoom Yousuf, Zubair Mushtaq, Syed Bilal, Mehraj Khan

**Affiliations:** 1 Cardiology, Sher-I-Kashmir Institute of Medical Sciences (SKIMS), Srinagar, IND; 2 Paediatrics, Sher-I-Kashmir Institute of Medical Sciences (SKIMS), Srinagar, IND

**Keywords:** microcatheter, neonate, obstructed tapvc, supracardiac, vertical vein stenting

## Abstract

Obstructive total anomalous pulmonary venous connection (TAPVC) is a life-threatening congenital anomaly requiring urgent intervention. A one-month-old, 2.5 kg neonate presented with tachypnea, respiratory distress, lethargy, and 70% oxygen saturation. Echocardiography revealed obstructed supracardiac TAPVC with a 20 mmHg gradient at the pulmonary venous confluence and a 3.5 mm atrial septal defect with right-to-left shunting. Due to the patient's critical condition, palliative stenting of the vertical vein (VV) was performed. Despite significant tortuosity, a Progreat microcatheter successfully crossed the obstruction. Angiography confirmed severe narrowing, and two overlapping stents (6×18 mm and 7×15 mm) were placed, reducing the gradient to 2 mmHg and improving oxygen saturation to 90%. Balloon atrial septostomy was also performed. While the neonate showed initial improvement, the condition worsened due to sepsis, and the infant expired two days later. This case highlights the feasibility of microcatheter-assisted VV stenting as a palliative measure in critically ill neonates with obstructed TAPVC and emphasizes the need for vigilant post-procedural care.

## Introduction

Supracardiac total anomalous pulmonary venous connection (TAPVC) occurs in 55% of cases of TAPVC [1]. The obstruction usually occurs due to the formation of bronchogenic vice as the vertical vein (VV) gets compressed anteriorly by the left pulmonary artery and posteriorly by the bronchus and descending aorta. The VV, which originates from the common chamber draining the pulmonary veins, typically courses anterior to the left pulmonary artery. However, in some cases, it may take a posterior path relative to the left pulmonary artery, making it vulnerable to compression between the artery and the left bronchus, especially when the pulmonary arteries are dilated, leading to dynamic and extrinsic obstruction. Although surgical correction is the standard form of treatment, transcatheter VV stenting can help in stabilizing such sick neonates before definitive surgery [2,3]. Literature describing these bridging procedures is limited but growing, with case reports and small series documenting the feasibility and short-term benefits of stenting in obstructed TAPVC. Microcatheter-assisted techniques, though not widely reported, offer distinct advantages in neonates due to the extreme tortuosity and narrow caliber of the venous structures involved. We describe one such case, which was complicated by tortuous anatomy of the vertical duct and was bailed out by the use of a microcatheter.

## Case presentation

**Figure 1:**
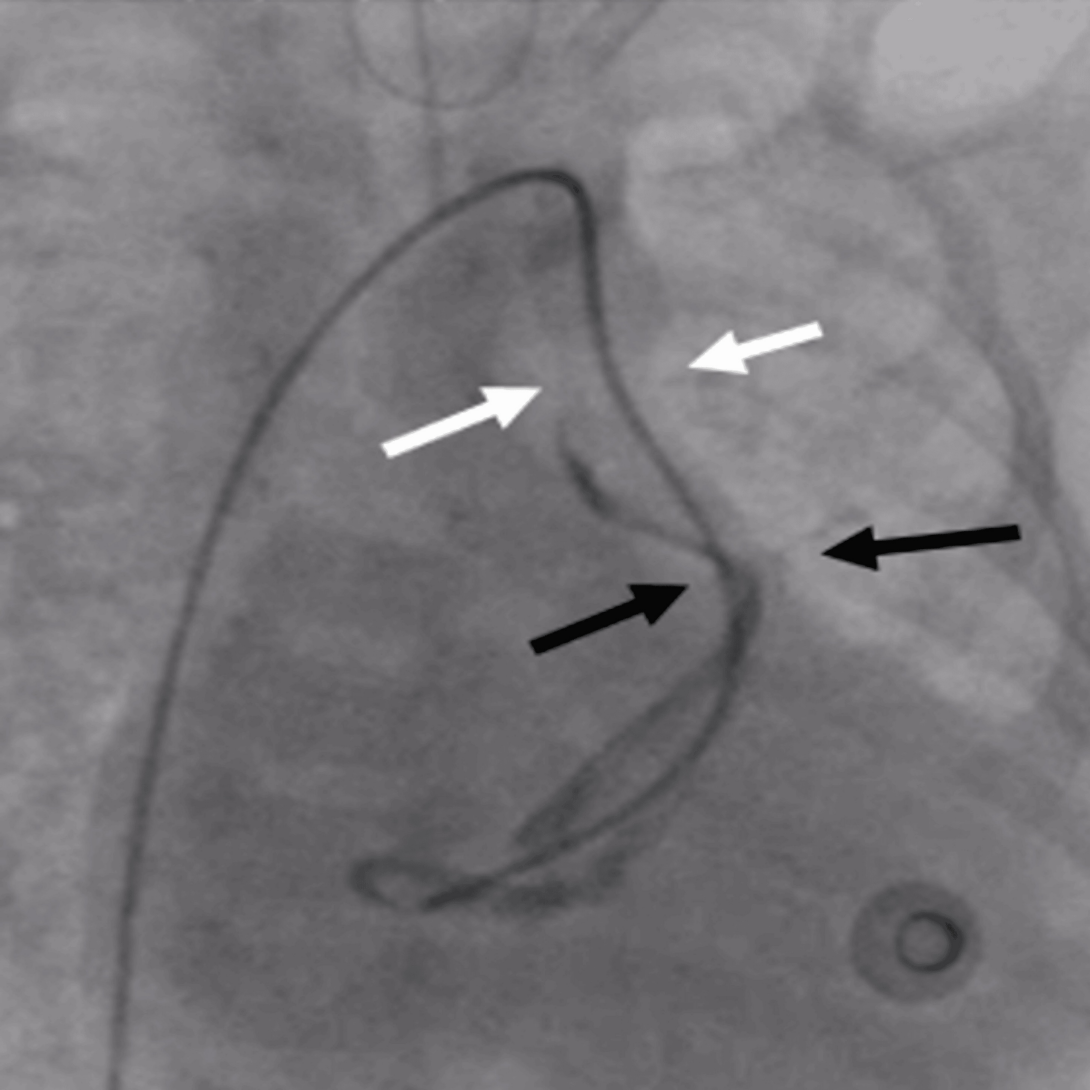
Angiogram with the help of a microcatheter showing a tortuous vertical vein with two sites of obstruction (black and white arrows)

A one-month-old, 2.5 kg neonate presented with tachypnoea, respiratory distress, and lethargy and was intubated elsewhere. He was shifted to our tertiary care center and on evaluation was found to have obstructed supracardiac TAPVC with obstruction at the level of PV confluence joining the VV with a mean gradient of 20 mmHg (normal gradient = 1-2 mmHg) and an atrial septal defect measuring 3.5 mm shunting right to left. The baby was sick and saturating at 70%. Given the patient's poor hemodynamic status, he was deemed high-risk for immediate surgical intervention. Following a multidisciplinary discussion with the pediatric cardiac surgical team, the decision was made to proceed with palliative VV stenting. Ultrasound-guided right femoral vein and left internal jugular access were taken with 6 French sheaths. Initial hemodynamics showed a mean pulmonary artery pressure of 60 mmHg (against systemic mean pressure of 65 mmHg). Initially, a 5 French JR 3.5 diagnostic catheter with 0.035 Terumo wire was taken to cross the VV into the common chamber. However, both Straight tip and J tip Terumo failed to cross. Finally, a 0.014 Whisper coronary wire could cross the VV into the common chamber and was parked deep into the right pulmonary vein. However, we could not track any catheter over the wire across the VV into the common chamber, including 4 French Multipurpose and 4 French JR catheters. The reason is probably tortuosity and tight obstruction of the VV. After trying multiple catheters, we were finally able to track the Progreat microcatheter into the common chamber. We took an angiogram with the microcatheter, which demonstrated severe narrowing of the VV (Figure 1).

**Figure 2:**
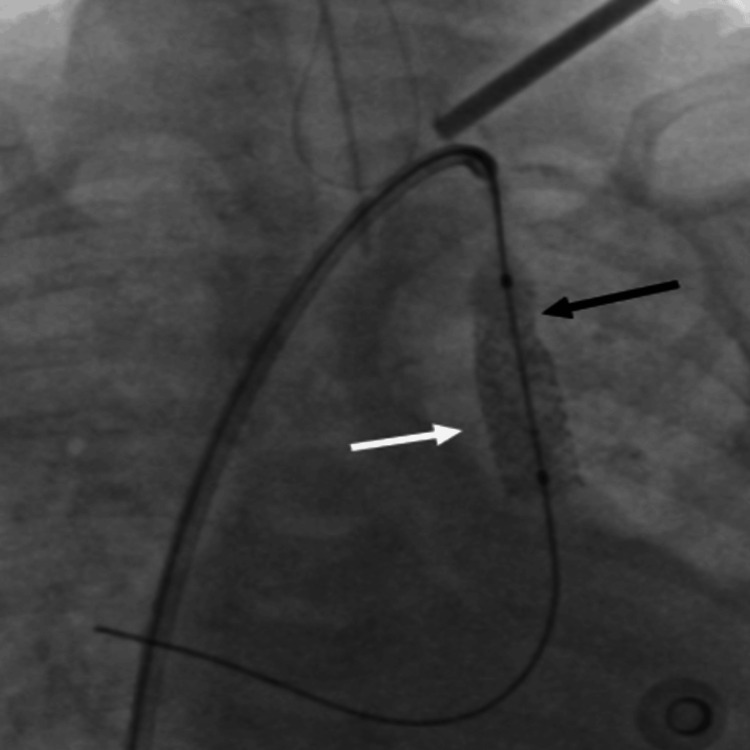
A 6 x 18 mm stent deployed in the vertical vein across the distal obstruction (the white arrow showing stent fully opened across the distal tight obstruction and the black arrow showing under expanded stent with residual obstruction at the upper end of the stent)

After that, the coronary wire was exchanged with the V 0.018 wire. The wire was passed deep into the right lower pulmonary vein. On the V 0.018 wire, we tracked six French RCA guides to the opening of VV into the innominate vein for support. We initially stented the tightest portion of VV with a 6 x 18 mm peripheral stent across the VV (Figure 2).

**Figure 3:**
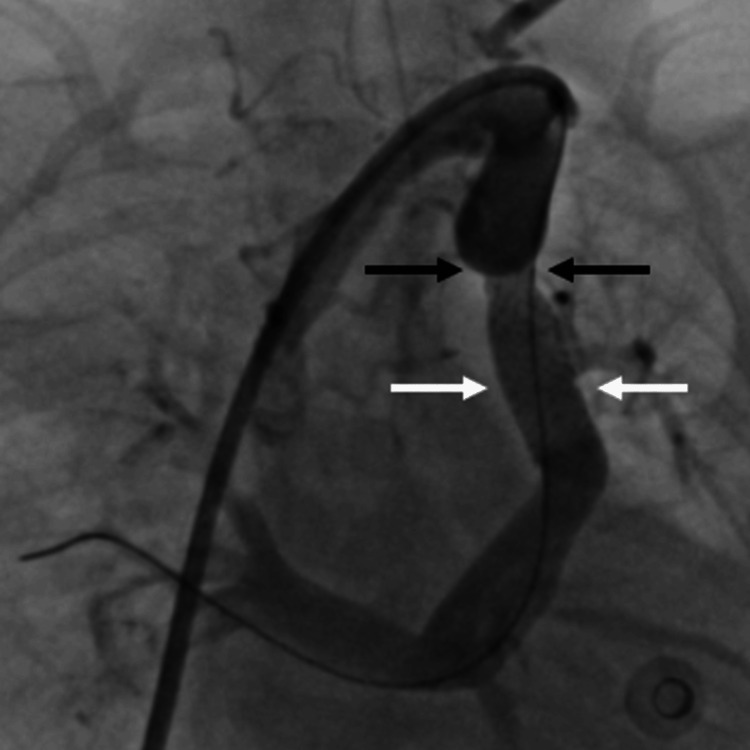
Angiogram showing good flow across the stent (the white arrow showing stent fully opened across the distal tight obstruction and the black arrow showing the under-expanded stent with residual obstruction at the upper end of the stent)

**Figure 4:**
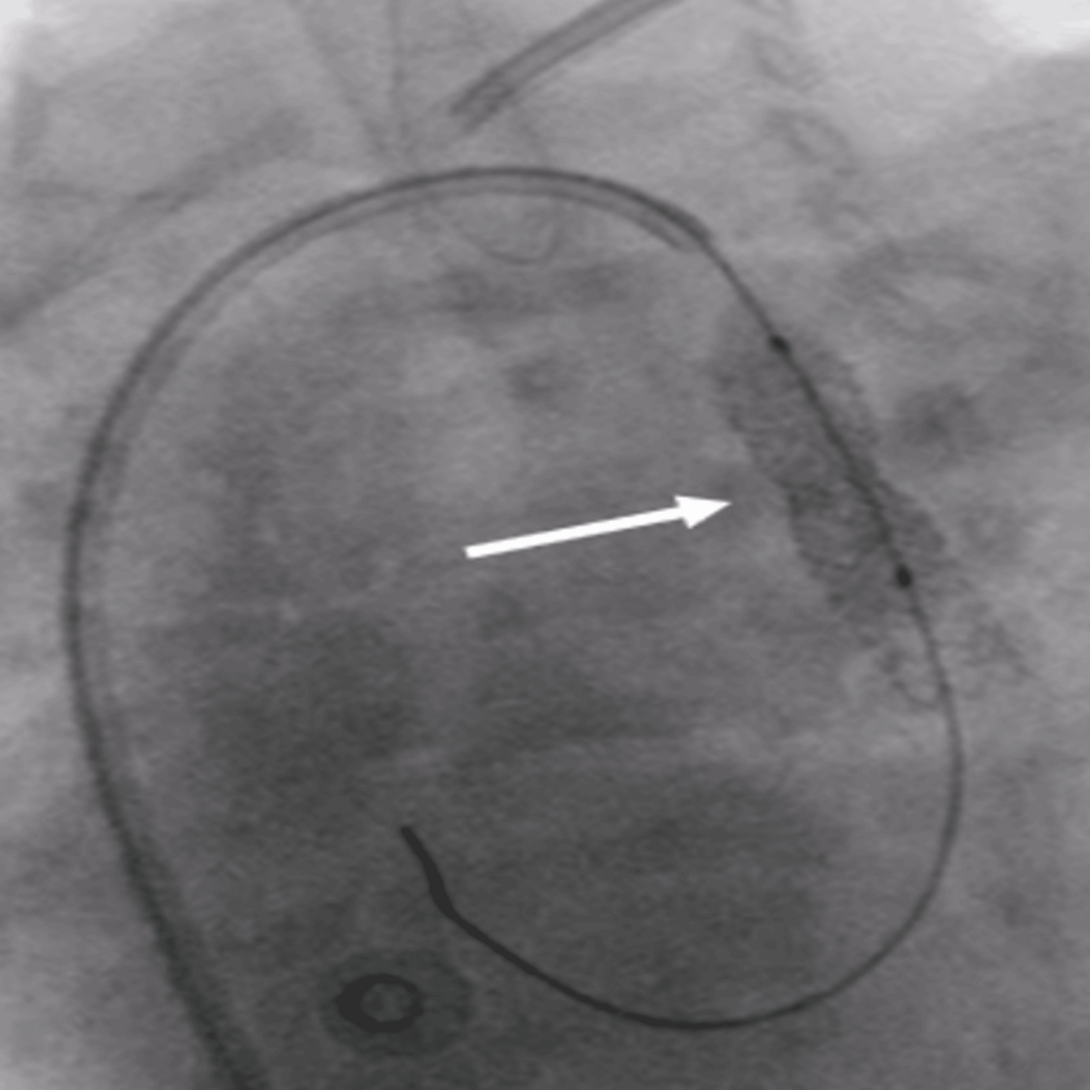
Fluoro image showing another stent 7x 15 mm being deployed proximally with the mild residual waist (white arrow)

The angiogram showed good flow across the stent; however, there was residual obstruction at the upper edge of the stent (Figure 3) so we stented with another stent proximally 7 x 15 mm (Figure 4).

Stent sizes were selected based on angiographic assessment of the VV diameter (approximately 6-7 mm), the length of the stenotic segment, and device availability. The 6×18 mm stent addressed the most narrowed portion. Residual proximal narrowing due to vessel tapering and angle required an additional 7×15 mm stent. Post stent, there was good venous outflow with a residual gradient of 2 mmHg. This was followed by balloon atrial septostomy (BAS) with a 10 Z septostomy balloon. BAS was performed as an adjunctive measure to enlarge the atrial septal defect, thereby enhancing systemic cardiac output and facilitating decompression of the pulmonary venous circulation to reduce pulmonary edema. Post stenting and BAS, the hemodynamics significantly improved with mean PA pressure falling to 20 mmHg, and oxygen saturation improved on the table to 90%. The hemodynamics pre- and post-procedure are shown in Table 1.

**Table 1:** Hemodynamic parameters before and after vertical vein stenting

Parameter	Pre-intervention	Post-intervention
Oxygen Saturation (%)	70%	90%
Pulmonary Artery Pressure (mean)	60 mmHg	20 mmHg
Systemic Arterial Pressure (mean)	65 mmHg	80 mmHg
Vertical Vein Gradient	20 mmHg	2 mmHg

The baby was extubated the following day, with echocardiography confirming good stent flow. On postoperative day 2, the neonate developed sepsis, with blood cultures growing Klebsiella pneumoniae, indicating a likely nosocomial source, possibly related to invasive lines and prolonged ICU stay. There were no signs of local infection at catheter insertion sites, and no procedural contamination was identified. Despite supportive care, the infant's condition deteriorated further due to sepsis, and the baby succumbed two days later.

## Discussion

We describe a case of VV stenting in a case of sick obstructive TAPVC neonate and highlight the use of microcatheters in navigating tortuous anatomy where usual catheters failed to cross the VV.

Although corrective surgery is the standard treatment for obstructive TAPVC, when surgery is high risk or not available [4], endovascular stenting of the VV can help as an effective bridging therapy [5-7]. Our baby was too sick with poor hemodynamic status and was considered high risk for any surgical intervention.

We took both femoral and jugular access. We initially tried to pass a 0.35 Terumo wire with RCA diagnostic catheter support to cross the VV into the common chamber. However, the Terumo wire failed to cross the VV, likely due to tortuosity and obstruction. We also attempted from the jugular access, but this was unsuccessful. Finally, a Whisper 0.014 coronary wire was able to cross into the common chamber and lower pulmonary vein. However, we were unable to negotiate any catheter over it to take angiograms and plan for stenting. We finally passed a Progreat (Terumo) microcatheter over the wire, which easily entered the common chamber. We were able to take an angiogram with the microcatheter and locate the site of obstruction. Through the microcatheter, we exchanged the coronary wire for a V 0.018 wire. Over the V 0.018 wire, we exchanged the diagnostic RCA catheter for a 6 Fr JR guide for more support, allowing us to pass the stents. After that, we were able to easily pass both stents over the V 0.018 wire.

Navigating the neonatal venous system is inherently challenging due to the small vessel caliber, extreme fragility of vascular walls, and frequent presence of sharp angulations, particularly in conditions like obstructed TAPVC. In our case, the VV exhibited significant tortuosity and narrowing, creating resistance points that prevented standard guidewires and catheters from crossing. Traditional 4-5 Fr catheters, though commonly used, lack the flexibility and trackability required to negotiate such anatomy without risking vessel injury or procedural failure. Microcatheters, by contrast, offer a smaller profile, superior torque control, and enhanced maneuverability, making them particularly advantageous in traversing tight curves and reaching distal targets in fragile neonatal vasculature. Recognizing this challenge early allowed for a targeted strategy using a Progreat microcatheter, which proved critical to the success of the intervention

We highlight the use of the microcatheter in this procedure, as it not only helped in navigating tortuosity but also in taking angiograms and completing the procedure successfully.

## Conclusions

This case highlights the technical challenges and potential benefits of microcatheter-assisted stenting of a tortuous, obstructed VV in a critically ill neonate with obstructed supracardiac TAPVC. We suggest the use of microcatheters in challenging anatomies, as they not only help in navigating complex structures but also assist in taking angiograms and facilitate the passage of hardware to complete the procedure successfully. Despite initial clinical improvement, the infant ultimately succumbed to sepsis, underscoring that while stenting can offer life-saving temporary relief, it is not without risk. This emphasizes the need for vigilant post-procedural monitoring and aggressive supportive care to mitigate complications in this vulnerable population. Future cases should consider early multidisciplinary involvement and proactive infection control to improve outcomes.
